# Current Role and Future Perspectives of Immunotherapy and Circulating Factors in Treatment of Biliary Tract Cancers

**DOI:** 10.7150/ijms.82008

**Published:** 2023-05-11

**Authors:** Simone Conci, Giovanni Catalano, Diletta Roman, Camilla Zecchetto, Eleonora Lucin, Mario De Bellis, Marzia Tripepi, Alfredo Guglielmi, Michele Milella, Andrea Ruzzenente

**Affiliations:** 1 Division of General and Hepatobiliary Surgery, Department of Surgical Sciences, Dentistry, Gynecology and Pediatrics, University of Verona, University Hospital G.B. Rossi, Verona, Italy.; 2 Digestive Molecular Clinical Oncology Research Unit, Section of Medical Oncology, University of Verona, University Hospital G.B. Rossi, Verona, Italy.

**Keywords:** biliary tract cancers, cholangiocarcinoma, immunotherapy, chemotherapy, checkpoint inhibitors, circulating factors, target therapy

## Abstract

Biliary tract cancers (BTCs) are a heterogenous group of malignancies arising from the epithelial cells of the biliary tree and the gallbladder. They are often locally advanced or already metastatic at the time of the diagnosis and therefore prognosis remains dismal. Unfortunately, the management of BTCs has been limited by resistance and consequent low response rate to cytotoxic systemic therapy. New therapeutic approaches are needed to improve the survival outcomes for these patients. Immunotherapy, one of the newest therapeutic options, is changing the approach to the oncological treatment. Immune checkpoint inhibitors are by far the most promising group of immunotherapeutic agents: they work by blocking the tumor-induced inhibition of the immune cellular response. Immunotherapy in BTCs is currently approved as second-line treatment for patients whose tumors have a peculiar molecular profile, such as high levels of microsatellites instability, PD-L1 overexpression, or high levels of tumor mutational burden. However, emerging data from ongoing clinical trials seem to suggest that durable responses can be achieved in other subsets of patients.

The BTCs are characterized by a highly desmoplastic microenvironment that fuels the growth of cancer tissue, but tissue biopsies are often difficult to obtain or not feasible in BTCs. Recent studies have hence proposed to use liquid biopsy approaches to search the blood circulating tumor cells (CTCs) or circulating tumor DNA (ctDNA) to use as biomarkers in BTCs. So far studies are insufficient to promote their use in clinical management, however trials are still in progress with promising preliminary results. Analysis of blood samples for ctDNA to research possible tumor-specific genetic or epigenetic alterations that could be linked to treatment response or prognosis was already feasible. Although there are still few data available, ctDNA analysis in BTC is fast, non-invasive, and could also represent a way to diagnose BTC earlier and monitor tumor response to chemotherapy. The prognostic capabilities of soluble factors in BTC are not yet precisely determined and more studies are needed. In this review, we will discuss the different approaches to immunotherapy and tumor circulating factors, the progress that has been made so far, and the possible future developments.

## Introduction

Biliary tract cancers (BTC) are rare and highly lethal epithelial cell malignancies that arise from the biliary duct cells. They represent the second most common primary liver malignancy after hepatocellular carcinoma, accounting for 15% of all primary liver tumors and 3% of gastrointestinal cancers 1. Incidence amounts to 10,000 new cases/year in Europe (0.5 to 3 cases per 100,000 people) and 12,000 new cases/year in the United States (1.6 cases per 100,000 people) 2,3. Incidence is higher in Asia, with 5.7 to 85 cases per 100,000 people 4,5. The main risk factors for BTCs are chronic viral infections (hepatitis virus B and hepatitis virus C), cirrhosis or non-alcoholic fatty liver disease, obesity, alcohol and tobacco consumption, diabetes, sclerosing cholangitis and liver fluke infections in endemic areas 6,7. Biliary tract cancers could be classified according to their anatomical site of origin in intrahepatic (iCCA), perihilar (pCCA), distal (dCCA) cholangiocarcinoma and gallbladder cancer (GBC). There are consistent reports of an increasing worldwide incidence of iCCA and a decreasing or stable incidence of extra hepatic CCA (both pCCA and dCCA) 8-14. Nowadays, surgery remains the only potential cure for these cancers, but post-surgery tumor relapses are common 15-17 resulting in poor prognosis. About 70% of patients are diagnosed at an advanced stage due to a lack of specific symptoms and screening protocols 18,19; for late-stage disease not suitable for radical resection, chemotherapy is a cornerstone for the treatment 20. The combination of cisplatin and gemcitabine [CISGEM regimen] is the standard first-line therapy for this kind of patients, with a median overall survival (OS) and a progression-free survival (PFS) of 11.7 and 8.0 months, respectively 21. The second-line chemotherapy, according to the ABC-06 phase 3 trial, is the combination of fluorouracil and oxaliplatin (FOLFOX regimen) 22; beyond second line there is no validated standard treatment. New therapeutic approaches are clearly needed to improve the survival outcomes for these patients. The main molecular pathways that characterize BTCs are the JAK/STAT signaling pathway, the FGF and Ras/BRaf/MEK/ERK pathway, the EGFR and HER2 signaling pathway, and many others. Farmacological therapies that target these altered signaling pathways are being tested in alternative to standard systemic therapy regimens as a way to improve the grim chanches of survival of patients with advanced BTCs. The main ongoing trials of these targeted therapies are summarized in *Table 1* 23).

## The immunotherapy revolution

Immunotherapy has changed the paradigm of cancer's treatment, leading the patient's immune system to attack cancer cells as though they were foreign invaders 24. Immune checkpoint inhibitors (ICIs) are up to now the most promising group of immunotherapeutic agents: they act by blocking the tumor-induced inhibition of the immune cellular response. Immune checkpoints are molecules that normally ensure self-tolerance, preventing the immune system from attacking cells indiscriminately. Moreover, ICIs are triggered when the receptor proteins on the surface of T cells recognize and bind to ligand mate proteins on other cells, such as tumor cells. These proteins are called immune checkpoint proteins. When these molecules connect with their partners proteins, they send an “off” signal to the T lymphocytes. This prevents the body's immune system from destroying cancer. Immune checkpoint inhibitors block checkpoint proteins from binding with their partner proteins. In this way there is no transmission of the “off” signal, so T cells are enabled to kill cancer 25.

The first ICI to be developed was *Ipilimumab*, an immune antibody that binds to CTLA4. When T cells meet an antigen-presenting cell (APC), two signals are required to stimulate T cell proliferation: the major histocompatibility complex (MHC) on APCs and the binding of CD28 on the T cell to the APC. The simultaneous activation of these signals induces proliferation of T cells and activates the expression of CTLA-4. As CTLA-4 accumulates in the T cell, it outperforms CD28 and disables T cell proliferation. By targeting CTLA-4, *Ipilimumab* shuts down one of the inhibitory pathways that block effective anti-tumor responses.

The other class of ICIs targets programmed cell death protein 1(PD-1) on T cells and its ligand (PD-L1) on the APCs. In particular, PD-1 downregulates T cell activation upon binding to PD-L1 on APCs. Blocking PD-1 or PD-L1 can, therefore, enhance the T cell response. The first anti-PD-1 therapies came into the clinic in 2014, with approval of *Pembrolizumab* and *Nivolumab* 26.

The other major class of immunotherapy is chimeric antigen receptor (CAR) T cells. In this therapy, T cells are removed from a patient and genetically engineered to express a CAR derived from a tumor-specific antigen; the CAR-expressing T cells, and their cytotoxic functions, are thereby specifiable directed toward tumor cells. The first CAR T cell therapy to be approved was *Kymriah* 24.

The ICIs are now approved for a variety of solid tumors including non-small cell lung cancer, melanoma, cutaneous squamous cell carcinoma, urothelial carcinoma, cervical cancer, Hodgkin's lymphoma, head and neck squamous cell carcinoma, Merkel cell carcinoma, renal cell carcinoma, MSI-H or dMMR colorectal cancer, and hepatocellular carcinoma 27.

The reduced immunogenicity of HCC along with the immunosuppressive tumor microenvironment of the liver has hampered the development and implementation of immunotherapy 28,29. Current immunotherapies for liver cancer include ICIs, the adoptive transfer of immune cells, bio specific antibodies, vaccines, and oncolytic viruses. The ICIs against PD-1 and CTLA-4 have demonstrated utility in HCC. In advanced CCA and GBC, PD-1 ICIs have resulted in antitumor responses, but only in a minority of select patients. Since the time ICIs were developed, there have been an increased percentage of patients eligible for this kind of therapy, from 1.5% in 2011 to 43.6% in 2018. Even the percentage of patients with cancer estimated to respond to checkpoint inhibitor drugs increased from 0.1% to 12.5% 30.

## State of the art and future developments of immunotherapy in BTCs

### Immune checkpoint inhibitors

**Figure 1:**
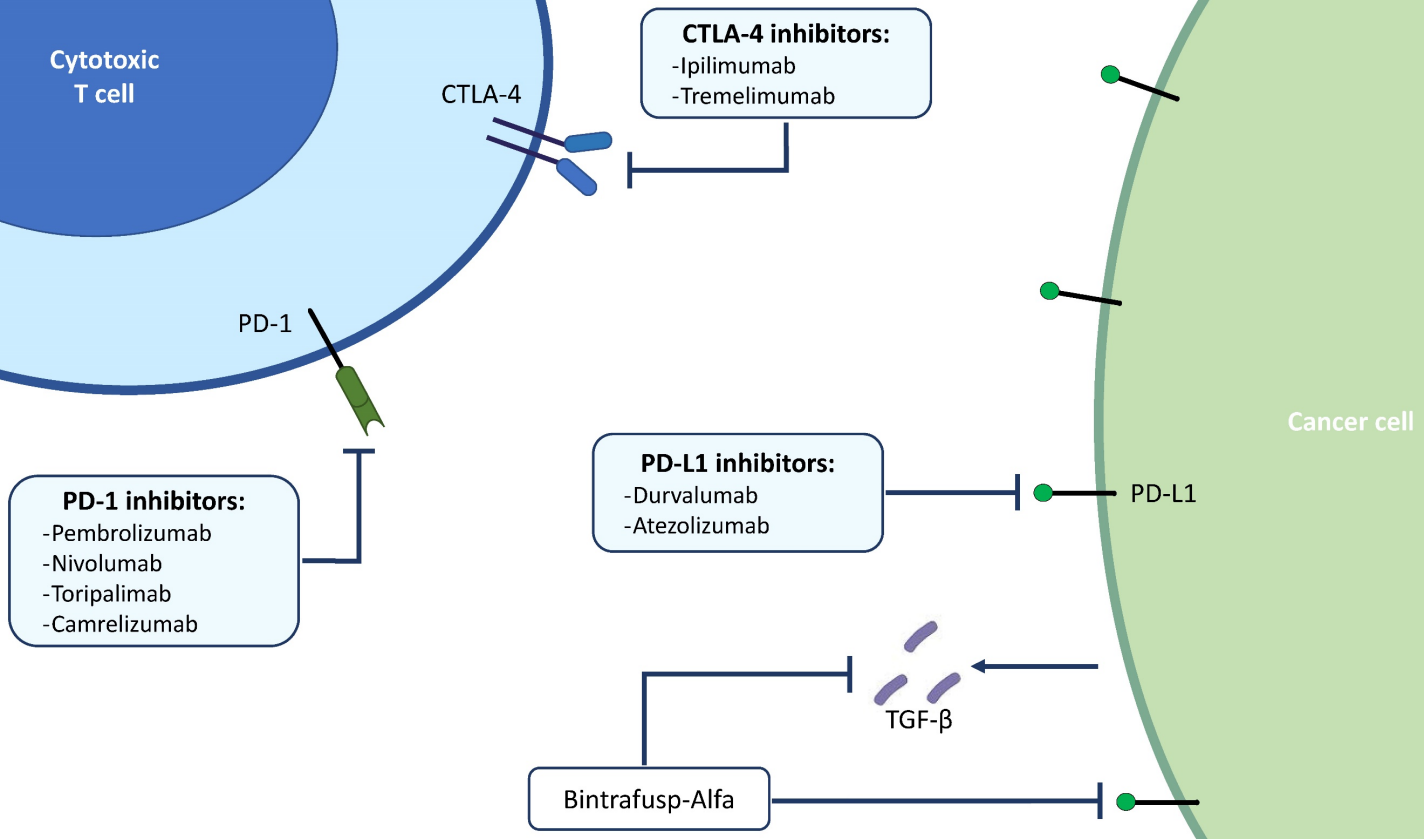
** Main targets of immunotherapy in BTCs.** BTCs; biliary tract cancers; PD-1, programmed death 1; PD-L1, programmed death ligand 1; CTLA-4, cytotoxic T-lymphocyte-associated protein 4; TGF-β, transforming growth factor beta.

Nowadays, the most widely studied therapeutic target for cancer immunotherapy is programmed death-ligand 1 (PD-L1) (*Figure 1*). It has been already reported how solid tumors with high level of microsatellite instability (also called “MSI-high”) have favorable responses to PD-L1 blockade immunotherapy 31,32. *Pembrolizumab*, which binds and blocks PD-1, is approved for many different solid tumors with microsatellite instability. The KEYNOTE-158 (phase 2) and KEYNOTE-028 (phase 1b) studies have evaluated the safety and efficacy of *Pembrolizumab* in advanced BTC: the monoclonal antibody showed manageable toxicity and durable antitumor activity in 6% to 13% of patients with advanced BTC, regardless of PD-L1 expression 33. Published results of the pivotal clinical trials that have tested immunotherapeutic drugs in BTCs are summarized in *Table 2*.

Thanks to these studies, *Pembrolizumab* was the first immunotherapeutic agent with an on-label indication for the treatment of BTC. It was approved by the FDA for treatment of a variety of advanced solid tumors, including BTC, that were MSI-high or mismatch repair deficient (dMMR), were not responsive to first line chemotherapy and had no satisfactory alternative treatment options. This was the first approval of a so-called “tissue-agnostic” anticancer treatment, which is based on the cancer's genetic and molecular features without regard to the cancer type or where the cancer started in the body 34. Unfortunately, the majority of BTCs are not MSI-high or dMMR. In fact, MSI-high tumors account for approximately 2% of BTCs, and not all MSI-high tumors respond favorably to ICI. Therefore, novel biomarkers are needed to screen for patients with BTC that could benefit from ICI therapy with PD-L1 blockade 35.

*Nivolumab*, another PD-L1 blocker, has also been tested both alone and in combination with gemcitabine and cisplatin (GEMCIS) in many phase 2 studies, with promising efficacy and manageable safety profile 36,37. In a phase 2 trial, Kim et al*.* enrolled 46 patients with advanced BTC who had done at least first-line chemotherapy. After the treatment with *Nivolumab*, the overall response rate (ORR) was 11%, progression free survival (PFS) was 3.7 months and overall survival (OS) was 14.2 months 37. Right now, the current National Comprehensive Cancer Network (NCCN) guidelines recommend Nivolumab as a subsequent-line treatment option for unresectable and metastatic BTC with disease progression, that are not MSI-high nor have high PD-L1 expression (class 2B recommendation) 38,39.

*Ipilimumab*, an anti-CTLA-4 drug, is currently undergoing clinical trials to test its efficacy on different solid tumors. In the CA209-538 (phase 2) clinical trial the combination of *Ipilimumab* plus *Nivolumab* was associated with positive outcomes in patients with advanced BTC. This combination has shown better response rates than single-agent anti-PD-1 therapy, with an objective response rate of 23% among patients with intra-hepatic and gallbladder cholangiocarcinoma. There were no responses in the patients with extrahepatic cholangiocarcinoma. Overall survival was 5.7 months (95% CI, 2.7-11.9 months) 40. Additional trials will be required to determine whether this combined immunotherapy regimen can be superior to single-agent anti-PD-1 therapy in patients with advanced BTC.

The efficacy of immunotherapy in BTC is being tested also as first-line therapy in chemotherapy-naïve patients. The KEYNOTE-966 phase 3 trial (NCT04003636) is currently underway and has enrolled patients with histologically confirmed diagnosis of advanced or unresectable BTC in order to evaluate the overall survival in patients who received GEMCIS + *Pembrolizumab* vs only GEMCIS therapy. Promising results from this phase 3 KEYNOTE-966 trial were recently announced: the combination of *Pembrolizumab* and GEMCIS demonstrated a statistically significant improvement in overall survival versus chemotherapy alone in this setting, although conclusive results have yet to be published 41. The TOPAZ-1 trial (NCT03875235), a double-blind placebo-controlled study of *Durvalumab* (an anti-PD-L1 drug) plus GEMCIS in chemotherapy-naïve patients with advanced BTC has recently ended. Overall survival (hazard ratio [HR] 0.80; 95%CI 0.66-0.97) and progression free survival (hazard ratio [HR] 0.75; 95%CI 0.64-0.89) were significantly improved in the group of patients who received *Durvalumab*, with manageable safety. The ORR was 26.7% in the group of patients receiving *Durvalumab* and 18.7% in the placebo arm 42. Based on the result from the TOPAZ-1 trial, *Durvalumab* is now approved by the Food and Drug Administration (FDA) for the treatment of adult patients with locally advanced or metastatic BTC in combination with GEMCIS chemotherapy. Furthermore, this regimen is now also recommended as first-line treatment in the NCCN guidelines as an option for first-line treatment of patients with unresectable, locally advanced, or metastatic disease 39.

*Bintrafusp-alfa* is a bifunctional protein composed of a human anti-PD-L1 IgG1 monoclonal antibody fused with two extracellular domains of the transforming growth factor β (TGF-β) receptor II (a TGF-β “trap”). *Bintrafusp-alfa* showed promising results in patients with BTC whose disease progressed after first-line treatment 43,44. The phase 2 INTR@PID BTC047 study evaluated *Bintrafusp-alfa* as a monotherapy in the second-line treatment in 159 patients with advanced or metastatic BTC who have failed or are intolerant of first-line platinum-based chemotherapy. The results showed an improved ORR of 10.1% (95% CI: 5.9% to 15.8%) and a manageable safety profile 45. Based on these results, *Bintrafusp-alfa* is currently under further investigation in patients with BTC, both as monotherapy and in combination with CISGEM. Ongoing trials for new immunotherapeutic agents are summarized in *Table 3*.

### Vaccine immunotherapy

Other types of immunotherapies, such as vaccines and cellular therapy, have been tested during the last years. A vaccine with four immunogenic peptides (HLA-A*2402-restricted epitope peptides, lymphocyte antigen 6 complex locus K, TTK protein kinase, insulin-like growth factor-II mRNA-binding protein 3 and DEP domain containing 1) was attempted by Aruga et al*.* The same group developed a phase 1 clinical trial of a multiple-peptide vaccination for patients with advanced BTC using three peptides: peptides-cell division cycle associated 1 (CDCA1), cadherin 3 (CDH3) and kinesin family member 20A (KIF20A). In both studies vaccines were well-tolerated but yielded very limited success 46,47. Other groups obtained very similar results using peptides such as MUC-1 (Mucin 1, cell surface associated) and WT1 (Wilms' tumor protein-1), with low to zero toxicity but very poor clinical response 48-50. As of now, the best strategy to develop a vaccine is to use several different antigenic peptides, although which to use seems to rely heavily on the different BTC subtype.

### CAR T cell theraphy

Chimeric antigen receptor (CAR) T cell therapy has already proven its efficacy and safety in patients with hematological malignancies such as relapsed lymphoblastic leukemia, diffuse large B-cell lymphoma, mantle cell lymphoma and others 51-53. CAR-T cell therapy is now being tested in many different solid tumors. Guo et al*.* in a phase 1 study evaluated the efficacy and safety of EGFR-specific CAR-T cells in patients with advanced unresectable or relapsed BTC. Of 17 evaluable patients, 1 achieved complete response and 10 achieved stable disease, and median PFS was 4 months (range, 2.5-22 months) 54. Feng et al. carried out a phase 1 clinical trial enrolling patients with HER2-positive advanced biliary tract and pancreatic cancers. The objective response rate was 55% while median PFS was 4.8 months (range, 1.5-8.3 months), showing the safety and feasibility of CAR-T-HER2 immunotherapy 55. Currently CAR-T cell therapy in BTC is still in its early development phase, but it seems to have encouraged overall response rate and manageable safety. Further studies are however needed to assess the long-term efficacy and to establish the correct subset of patients with BTC that would benefit from this kind of therapy.

### Innate immune cells therapy

Recent clinical studies have highlighted the possible role of innate immune cells as potential candidates for new immunotherapeutic strategies. Natural killer cells (NK) are especially known for their ability to destroy tumor cells *in vitro*, determined by the balance of inhibitory and activating receptors on their cell surface. However, it is technically difficult to generate large numbers of NK cells and they show a short life span *in vivo*. The clinical application of NK cells is carried out by culturing and activating the NK cells isolated from blood of either patient (autologous) or blood donor (allogeneic). While positive clinical outcomes were observed in hematological cancer patients, the transfer of expanded autologous NK cells has not yet shown clear positive clinical responses in solid tumors. Recently, NK cell therapy for cholangiocarcinoma has been successfully done (NCT03358849) with allogeneic NK cell, and further studies are ongoing (NCT03937895). The possibility to engineer CAR-NK cells has attracted much attention, but further studies are needed to assess feasibility and outcomes in patients with BTC 56.

## Predictive and prognostic biomarkers in BTCs

### Current markers for ICI therapy response

In *Table 4* we have summarized current available markers for ICI therapy response in BTCs. Programmed death ligand 1 (PD-L1) is the transmembrane molecule that binds to PD-1 in the process of immune checkpoint inhibition. The ICI drugs like *Pembrolizumab* bind PD-1 to stop the tumor-induced immune suppression. Thus, it was though that assessing the tumor tissue's expression levels of PD-L1 could hint to patients' response to treatment with ICI (57.58). However, the role of PD-L1 expression in BTC is still to be precisely assessed. As previously stated, the KEYNOTE-158 and KEYNOTE-028 trials in patients with advanced BTC did not found any correlation between PD-L1 levels assessed with immunohistochemistry and response to ICI treatment with *Pembrolizumab* 33. In a phase 2 trial of *Nivolumab* in patients with advanced BTC already treated with first line chemotherapy conducted by Kim and colleagues, patients with ≥1% of tumor cells expressing PD-L1 had a statistically significant higher median PFS compared with patients with PD-L1-negative tumor tissue 37. Data regarding assessment of PD-L1 in order to initiate ICI therapy is still controversial. Unfortunately, several methodological issues are at fault in the evaluation of PD-L1, namely the use of different assays, the differences in scoring systems, and the discrepancy between metastatic and primary lesions. These issues could contribute to the discording results reported in the literature 59.

The overall number of somatic mutations in a tumor cell is referred as “tumor mutation burden” (TMB). Given that more mutations a tumor has, the more immunogenic peptides will be generated and displayed on the major histocompatibility complex (MHC) on the tumor cell surface, it is though that tumors with high TMB will be more immunogenic. In fact, therapies with ICI showed at first improved outcomes in tumors with typically high TMB, such as non-small cell lung cancer and melanoma 60.

The gold standard assessment method for TMB is currently whole-exome sequencing (WES). This method has been used in several studies, but it is not feasible in clinical practice because of its high cost and long turnaround time. Therefore, TMB is routinely assessed on tumor-biopsies using panel-based sequencing, but differences in panel size, mutation types, and bioinformatic platforms exist, thus making this process not standardized. Furthermore, WES covers approximately 30 Mb of coding regions, while panel-based sequencing targets different genes, depending on which panel it is used, usually between 0.80 and 2.40 Mb 60,61. In the KEYNOTE-158 trial, tumors with a TMB greater than 10 mutations per megabase (mut/Mb) were considered “TMB-high” and showed better efficacy of the *Pembrolizumab* immunotherapy. Unfortunately, in this same study, none of the 63 patients with BTC had a TMB greater than 10 mut/Mb 62. In a genomic profiling study of 309 tumor biopsies from patients with BTC, *Jain et al.* found that only 60 patients had a TMB greater than 6 mut/Mb, of which 9 patients had a TMB greater than 20 mut/Mb 63. In a recent study by Zhang et al*.* on 24 patients with BTC, 3 patients with TMB-high were treated with ICI and achieved response to therapy (2 partial responses and 1 complete response) 64. Thanks to the KEYNOTE-158 trial *Pembrolizumab* is currently approved by the FDA for patients with any advanced solid tumor, including BTC, with a TMB greater than 10 mut/Mb. However, while high-TMB has been associated with improved survival in patients receiving ICI in a wide variety of cancer types 65,66, further studies are needed for the validation of its predictive capabilities in clinical practice. Issues remain regarding the optimal TMB cutoff, which could potentially vary between different solid tumors, and the best testing platforms 60. The data produced on TMB used as a marker for ICI therapy in BTC has been so far not conclusive, given the low rates of TMB-high BTC. Thus, further studies are needed to assess its potential predictive value.

Tumors that have a deficiency in the mismatch repair mechanism (mismatch repair deficiency, dMMR) tend to develop many more mutations than other kinds of tumors, therefore the neoantigens generated from these mutations make these tumors more immunogenic. Tumors that have dMMR are generally characterized by microsatellite instability (MSI) 19. As stated before, solid tumors with MSI have showed good response to therapy with ICI 31,32. Thanks to the KEYNOTE-158 trial, the FDA has already approved *Pembrolizumab* for treatment of a variety of advanced solid tumors, including BTC, that had MSI or dMMR, that were not responsive to first line chemotherapy and had no satisfactory alternative treatment options 33. Despite MSI being a good predictor of good response to immune checkpoint blockade, MSI-high BTC seem to be quite rare. In the patient cohort studied by Goeppert and colleagues the number of patients with high level MSI (MSI-high) BTC was about 2%. Even with this low rate of MSI-h BTC, they suggest testing for MSI especially in young patients showing with an atypical histomorphology 35. The data we have so far is not enough to implement routine MSI screening in BTC patients. Anyway, the evaluation of this biomarker in concert with other ones could provide us with a perfect tool to assess prognosis and predict treatment responses in BTC patients 59.

### The role of circulating factors in BTCs

Cholangiocarcinoma is characterized by a highly desmoplastic microenvironment containing stromal cells, especially cancer-associated fibroblast (CAFs), and other immune cells such as tumor-associated macrophages (TAMs), tumor-infiltrating lymphocytes (TILs) that release many cytokines and chemokines that stimulate cancer growth and recruitment of other immune cells 67,68.

Chemokines are a family of small proteins that attract leukocytes and are involved in tumor genesis. For instance, in cholangiocarcinoma, CCL2 produced by CAFs 69, leads to tumor progression, modulating metastasis, angiogenesis and cancer proliferation 70.

Many other soluble factors, including MCP-1, CXCL-12, CXCL-14, PDGF, TGFb, FGF1/2, are responsible for the persistent activation of CAFs; these cells stimulate tumor growth by secreting other soluble factors 71. Chuaysri et al*.* 72 demonstrated that patients with high levels of CAFs have the worst prognosis. Finally, CAFs contribute to recruit inflammatory cells, maintaining the tumor microenvironment neoangiogenesis 73.

Neoplastic cells release CCL-2 that activates CAFs and TAMs to become Treg, creating an immunosuppressive environment by secreting TGF β and IL-10 74,75. Tregs overexpress FoxP3, a transcription factor associated with the up-regulation of CTLA-4 on the cell surface; CTLA-4 binds to CD80 expressed by antigen-presenting cells and inhibits cytotoxic T-cell activation, contributing to tumor development 76. FoxP3, a distinctive feature of Tregs, is overexpressed also by CCA cells, thus correlating with lymphatic metastasis and poor survival 77,78. Knockdown of FoxP3 in CCA cells *in vitro* reduced proliferation and invasiveness, inhibited T cell survival, and reduced IL10 and TGF-β signaling in the tumor microenvironment 78.

### Circulating Cells

The analysis of tumor biomarkers isolated from biological fluids, referred as “liquid biopsy”, originated as a way to search for circulating tumor cells (CTCs). These CTCs are shed from the primary tumor site and then circulate through the bloodstream. Studies have searched the possibility to use these CTCs both as a non-invasive way to perform a tumor biopsy and to diagnose the presence of minimal residual disease undetectable by high-resolution imaging technologies. Data from CTCs could also be useful to understand the various mechanisms of drug resistance, tumor progression and metastasis 79. The first step to CTC isolation involves the so-called enrichment process, a selection based on biological or physical characteristics. Immunoselection, the main biological enrichment process, selects CTCs based on the detection by antibodies of specific markers, mostly epithelial cell-adhesion molecules (EpCAM) but also mesenchymal proteins 80,81. Studies on CTCs in BTC are limited to EpCAM-enriched CTCs have shown how CTCs seem to be associated with tumor extent and overall survival in patients with BTC 82-84. In a recent study, Reduzzi et al. confirmed the prognostic role of eCTCs on survival. However, they could not find any association with other kinds of CTCs that expressed different membrane markers 85.

Other protein expressed linked to the epithelial-mesenchymal transition (EMT) have been analysed in CTCs to search for a correlation with cancer progression. In a prospective clinical trial by Han et al. Vimentin-positive CTCs (V-CTCs) were analysed besides eCTCs in a sample of 62 patients, of which 52 had BTC and 10 had benign biliary diseases. In this study, the two groups did not show any statistically significant difference in CTC and V-CTC count. However, a statistically significant difference was found by analysing the V-CTC/total CTC count ratio (VCR). Furthermore, a blood concentration of V-CTC higher than 50/mL was correlated with survival rates, thus pointing V-CTC out as a potential biomarker for early diagnosis and prognosis in BTC 86.

### Circulating DNA

Through the so-called “liquid biopsy” it is possible to search for circulating free DNA (cfDNA), circulating tumor DNA (ctDNA) and circulating cell-free RNA (ccfRNA) to scan for possible tumor-specific genetic or epigenetic alterations. This kind of analysis is fast, non-invasive, cost-effective, and could represent a way to achieved early diagnosis of BTC, monitor tumor response to treatment, and detect tumor recurrences in advance. Furthermore, the process can be repeated multiple times to track the tumor's genetic changes over time in a non-invasive way during follow-up. Since the discovery of foetal cfDNA in the maternal bloodstream, cfDNA has become a popular research topic. The majority of cfDNA is released in the blood stream by lysis of normal cells. A small portion of it, called ctDNA, comes directly from primary tumor cells, metastatic cells, or CTCs 87,88. Recent studies have suggested concordance rates between tumor and plasma samples from 80% to 90% 89. In BTC, ctDNA analysis could play an even more important role, since biopsy samples are often inadequate for molecular profiling in CCA and GBC 90. Mody et al*.* enrolled 124 patients with BTC who underwent ctDNA testing with a 73-gene panel. Excluding variants of unknown significance, alterations were observed in 55% of patients, mainly *BRAF* mutation, *ERBB2* amplification, *FGFR2* fusions and mutations, and *IDH1* mutations. The technique was shown to be feasible in BTC, however the concordance between ctDNA and tissue biopsy has to be precisely assessed 91. In a phase 2 study, Jensen et al. analysed ctDNA from 24 patients with *KRAS*-mutated BTC. In 13 of 24 patients the known tumor tissue KRAS alteration was detectable at baseline. Patients with detectable *KRAS* mutation in ctDNA showed inferior PFS and OS. Furthermore, survival was significantly improved in patients with low level of total plasma DNA at baseline 92. Chen et al*.* used next generation sequencing of 150 cancer-related genes to detect gene alterations in blood ctDNA from 154 patients with BTC. *TP53* was the most frequently altered gene in ctDNA, followed by *KRAS* and *EGFR*. For most genes, the mutation frequencies in ctDNA were similar with those detected in tissue samples. This study outlines the role for ctDNA as a potential complementary tool in the clinical practice, aiding to screen patients who may benefit from targeted therapies 93. In a prospective study, Csoma et al. compared 25 tumor biopsy and 25 paired liquid biopsy samples from patients with BTC. Their analysis found a positive significant correlation between estimated tumor volume and the quantity of cfDNA. Pathogenic variations in the genetic material were proven in 68% of patients and presented in some of the most usually affected genes in BTCs such as *FGFR2*, *IDH1*, *IDH2*, *KRAS* and *TP53*, and most of them were matched between ctDNA and tissue biopsies 94. The consistency between blood ctDNA and tumor biopsy analysis emerged in the latest years could be the key to a new perspective for BTCs personalized therapy. As of now, however, more studies are needed to link ctDNA to survival outcomes and treatment response.

In BTC, the possibility to search for ctDNA not only in blood but also in the bile has recently emerged as a valid alternative. In a study on 42 patients with BTC there was an 80% mutational concordance between the paired bile ctDNA and tumor biopsy sample, giving the proof-of-concept that ctDNA on the bile of BTC patient could be an effective approach to genetic characterization in this subset of patients 95.

Some studies proposed DNA-methylation patterns as a tool to differentiate patients with CCA from healthy controls in a less invasive way. Assessing DNA-methylation patterns in serum cfDNA, bile cfDNA or biliary brushing samples has been already tested in many trials. Although several genes have been reported to be methylated in BTC, statistical data is not sufficient yet to determine which one is a potential biomarker for cancer detection 96-98.

A study by Qiu et al*.* identified 3369 common differentially methylated regions (DMRs) in 105 patients with BTC. A lower level of methylation was associated with a remarkably longer overall survival (hazard ratio [HR] = 0.07, p=0.017). Furthermore, BTCs with minimal methylation changes had infiltration of CD8+ lymphocytes, and PD-L1 expression, indicating an inflamed tumor immune microenvironment with PD-L1 expression elicited by immune attack, potentially suggesting better immunotherapy efficacy. More studies are needed to test immunotherapy efficacy combined with demethylation agents 99.

Besides DNA, some studies have highlighted the role of non-DNA molecules as possible biomarkers in BTCs. Non-coding RNA (ncRNA) is a kind of RNA that will not be translated into proteins. It can be detected in the blood, and it has been extensively studied for diagnostic purposes 100. MicroRNA or miRNA is the preferred marker among all the various types of ncRNAs because of its more stable structure, making it a more reliable marker than the other ncRNAs 101,102. A study by Kishimoto et al. aimed at evaluating whether circulating miR-21 could be a potential biomarker for BTC, analysed plasma samples from 94 patients with histologically proven BTC, 23 patients with benign biliary disease, and 50 healthy volunteers. Expression levels of miR-21 were significantly higher at baseline in patients with BTC and decreased significantly after surgery. The use of a combination of plasma miR-21 and CA19-9 levels to differentiate BTC patients from healthy volunteers or patients with benign biliary disease appeared very promising: in this study, miR-21 was a highly sensitive biomarker, while CA19-9 had a high specificity 103. Besides, miR-21 is not a specific biomarker for BTC and has been reported as a biomarker in other types of cancers such as lymphoma, colorectal cancer, oesophageal squamous cell carcinoma and hepatocellular carcinoma 104-107.

Assessing miRNA not only on the blood but also on the bile has also emerged as a valid alternative. In a study by Shigehara et al*.*, bile was sampled from 9 patients with BTC and 9 age-matched patients with choledocholithiasis. The presence and stability of miRNAs on these bile samples was confirmed. Furthermore, differential analysis demonstrated that 10 of the 667 miRNAs analysed were significantly more highly expressed in the malignant group than in the benign group, and the ROC-curve analysis showed that some specific miRNAs, namely miR-9 and miR-145, could be useful diagnostic markers for BTC 108.

## “Hot” and “Cold” tumors

It is well known how the heterogeneity of the tumor microenvironment (TME) has a role in determining how well a tumor responds after immunotherapy. Non-T cell-infiltrated tumors, the so-called “cold” tumors, contain very few CD8+ T cells but harbor many immunosuppressive cells such as M2-like TAMs (tumor-associated macrophages), MDSCs (myeloid-derived suppressor cells) and tolerogenic DCs (dendritic cells). These tumors have a subpar response to immunotherapy in contrast with the so-called “hot” tumors, which have a high concentration of CD8+ T cells and express more immune checkpoint molecules and have therefore a good response to immunotherapy. As of now, many studies are designing ways to improve the efficacy of checkpoint inhibitors in “cold” tumors by turning them into “hot” tumors through the activation of a pro-inflammatory process. Combining immune checkpoint inhibitors with targeted therapy or with radiotherapy, traditional systemic therapy, or other agents (such as cell-based therapies, vaccines, cytokines, or colony stimulating factors) could improve the efficacy of immunotherapy and increase drastically the number of patients that could benefit from it 109. Furthermore, many of the biomarkers that have been studied to predict treatment response in BTCs are ultimately immune-related biomarkers (such as PD-L1 expression): therefore, these markers could be unreliable in “cold” tumors. Given this apparent pivotal role of the TME, the careful assessment of the immunological status of the tumor could be used as a marker itself to differentiate between “hot” and “cold” tumors. However, maintaining a strict distinction of “hot” versus “cold” tumors could prove itself really dangerous as it turns an overly complex scenario into a “black or white” categorization 110.

## Conclusions

Immunotherapy is changing the approach to the treatment of cancer; so far, immune checkpoint inhibitors are approved as second line therapy in BTCs, for patients with MSI instability, TMB-high and PDL-1 overexpression, not responsive to first-line chemotherapy. However, ongoing studies suggest promising results of ICIs in a wider subset of patients, alone and in combination with standard chemotherapy. Moreover, circulating factors such as tumor DNA, could represent a new tool to achieve early diagnosis of BTC, monitor tumor response to treatment and predict tumor recurrence.

## Figures and Tables

**Table 1:** Main ongoing trials for targeted therapy in BTC.

Study name	Phase	Drug / Target	Setting
FIGHT-302 (NCT03656536)^111^	3	Pemigatinib / FGFR2	Unresectable or metastatic BTC with FGFR2 rearrangement who did not underwent prior systemic therapy.
PORCUPINE 2 (NCT04907851)^112^	2	Denosumab / RANKL RXC004 / Porcupine	PDAC and BTC which have progressed after first line of SoC systemic therapy.
NCT04211168^113^	2	Lenvatinib / VEGFR	Histologically confirmed advanced BTC who progressed after first line systemic therapy.
FIDES-01 (NCT03230318)^114^	2	Derazantinib / FGFR2	Histologically confirmed advanced BTC with confirmed FGFR2 fusion status, who progressed after at least one regimen of systemic therapy.
My Pathway (NCT02091141)^115^	2	Trastuzumab / HER2 Pertuzumab / HER2	Histologically confirmed metastatic solid tumors who have already received standard first-line systemic treatment and have HER2 overexpression or amplification.
NCT04238715^116^	2	E7090 / FGFR2	Histologically confirmed unresectable iCCC or phCCC, with centrally confirmed FGFR2 gene fusion by FISH, who received at least one prior line of systemic therapy.

BTC: biliary tract cancer, PDAC: pancreatic ductal adenocarcinoma, SoC: Standard of care, iCCC: intrahepatic cholangiocarcinoma, phCCC: peri-hilar cholangiocarcinoma, FISH: fluorescent in-situ hybridization, FGFR2: fibroblast growth factor receptor 2, RANKL: Receptor Activator of Nuclear Factor κ B ligand, VEGFR: vascular endothelial growth factor receptors, HER2: human epidermal growth factor receptor 2.

**Table 2:** Published results of main clinical trials for immunotherapy in BTC.

Study name	Phase	Drug / Target	Setting	Outcomes
KEYNOTE-028^33^	1b	Pembrolizumab / PD-1	Histologically confirmed advanced BTC, with disease progression after ≥1 prior standard therapy.	ORR 13% PFS 1.8 months OS 5.7 months
KEYNOTE-158^33^	2	Pembrolizumab / PD-1	Previously treated unresectable or metastatic MSI-H/dMMR non-colorectal cancer, including advanced BTC.	ORR 5.8% PFS 2 months OS 7.4 months
NCT02829918^37^	2	Nivolumab / PD-1	Histologically confirmed advanced refractory BTC undergoing treatment with 1-3 lines of systemic therapy.	ORR 22% PFS 3.68 months
CA209-538^40^	2	Nivolumab / PD-1 Ipilimumab / CTLA4	Unresectable or metastatic rare cancers, including advanced BTC.	ORR 23% PFS 2.9 months OS 5.7 months
TOPAZ-1 (NCT03875235)^44^	3	GEMCIS + Durvalumab / PD-L1	Chemotherapy-naïve patients with advanced BTC.	ORR 26.7%
INTR@PID BTC047 (NCT03833661)^45^	2	Bintrafusp-alfa / PD-L1:TGF-β	Second-line treatment in patients with advanced or metastatic BTC who have failed or are intolerant to first-line platinum-based chemotherapy.	ORR 10.1% PFS 1.8 months OS 7.6 months
NCT01938612^118^	1	Durvalumab / PD-L1 Tremelimumab / CTLA4	Second line treatment for advanced or metastatic solid tumors.	ORR 10.8% OS 10.1 months
JapicCTI-153098^119^	1	GEMCIS + Nivolumab / PD-1	First line treatment of unresectable BTC.	ORR 37% PFS 4.2 months
NCT03311789^120^	2	GEMCIS + Nivolumab / PD-1	First line treatment of unresectable BTC.	ORR 55.6% PFS 6.1 months OS 8.5 months
NCT01869166^54^	1	CART / EGFR	EGFR‑positive (>50%) advanced unresectable, relapsed, or metastatic BTC.	PFS 4 months
NCT01935843^55^	1	CART / HER2	HER2-positive (>50%) advanced unresectable, relapsed, or metastatic BTC and pancreatic cancers.	ORR 55% PFS 4.8 months

BTC: biliary tract cancer, ICIs: immune checkpoint inhibitors, PD-1: programmed cell death protein 1, PD-L1: programmed death-ligand 1, CTLA-4: cytotoxic T-lymphocyte antigen 4, GEMCIS: gemcitabine + cisplatin chemotherapy, ORR: Overall response rate, PFS: progression free survival, OS: overall survival, MSI-H: high microsatellite instability, dMMR: mismatch repair deficiency, CAR-T: chimeric antigen receptor T cells, EGFR: epidermal growth factor receptor, HER2: human epidermal growth factor receptor 2, TGF-β: Transforming growth factor beta.

**Table 3:** Main ongoing trials for immunotherapy in BTCs.

Study name	Phase	Drug / Target	Setting
Keynote - 158 (NCT02628067)^33^	2	Pembrolizumab / PD-1	Histologically/cytological confirmed incurable advanced BTC, disease progression after ≥1 prior standard therapy, ECOG-PS 0-1, no prior exposure to ICIs.
CA 209-538 (NCT02923934)^40^	2	Ipilimumab / CTLA-4 + Nivolumab / PD-1	First or second line therapy in neuroendocrine tumors, rare gynaecological tumors and advanced upper GI tumors, including BTC.
Keynote-966 (NCT04003636)^117^	3	GEMCIS + Pembrolizumab /PD-1	First line therapy for advanced or unresectable BTC.
NCT03797326^121^	2	Lenvatinib / VEGFR + Pembrolizumab / PD-1	Second line therapy in selected solid tumors, including BTC.
NCT04720131^122^	2	Capecitabine + Camrelizumab / PD-1 + Apatinib / VEGFR2	First or second line treatment for advanced BTC.
NCT04708067^123^	1	RT + Bintrafusp-alfa/PD-L1:TGF-β	Second line treatment for advanced or metastatic iCCC.

PD-1: programmed cell death protein 1, BTC: biliary tract cancer, ICIs: immune checkpoint inhibitors, ECOG-PS: eastern cooperative oncology group- performance status, PD-L1: programmed death-ligand 1, CTLA-4: cytotoxic T-lymphocyte antigen 4, GI: gastrointestinal, GEMCIS: gemcitabine + cisplatin chemotherapy, RT: radiation therapy, iCCC: intrahepatic cholangiocarcinoma, TGF-β: Transforming growth factor beta.

**Table 4:** Current available biomarkers for BTCs.

Biomarkers	Available data
**PD-L1**	Controversial data: - Keynote 158^33^ and Keynote 028^33^: no correlation between PD-L1 levels and response to ICIs; - NCT02829918^37^: patients with ≥1% of tumor cells expressing PD-L1 had a higher median PFS compared with patients with PD-L1-negative tumor tissue.
**TMB**	No sufficient data, given the low rate of TMB high BTCs: - Keynote 158^33^: tumors with TMB high showed better efficacy of Pembrolizumab. None of the patients with BTCs were TMB high.
**MSI**	MSI is a good predictor of response to ICIs; however, MSI-high BTCs are rare. - Keynote 158^33^: Pembrolizumab for treatment of BTC that had MSI or dMMR.
**EpCAM-enriched CTCs**	- Reduzzi et al.^85^ confirmed the prognostic role of eCTCs on survival in BTCs.
**V-CTCs**	- Han et al^86^: V-CTC > 50/mL blood is a significant factor affecting survival in patients with BTCs.
**ctDNA**	Potential complementary tool in the clinical practice to detect gene alterations, aiding in screening patients who may benefit from targeted therapies. - Chen et al^93^: for most genes, the mutation frequencies in ctDNA were similar with those detected in tissue samples. - Csoma et al^93^: positive correlation between the estimated tumor volume and cfDNA yield; Comparing tissue and LB results, similar tumor variant burden was observed.
**ncRNA (eg: miRNA)**	- Kishimoto et al^103^: increased level of miR-21 in patients with BTCs, making it a highly sensitive biomarker.

PD-L1: programmed death-ligand 1, ICIs: immune checkpoint inhibitors, PFS: progression free survival, TMB: tumor mutational burden, MSI: microsatellite instability, dMMR: mismatch repair deficiency, EpCAM: epithelial cell adhesion molecule, CTC: circulating tumor cells, V-CTC: vimentin-positive CTC, ctDNA: circulating tumor DNA, LB: liquid biopsy, cfDNA: circulating free DNA, ncRNA: non coding RNA, miRNA: micro-RNA.
